# Supplementary material for: Implicit bias in safety-aligned large language models: A multi-faceted evaluation of clinical decision-making and health equity
Source: PLoS One. 2026 May 19;21(5):e0348819. doi: 10.1371/journal.pone.0348819 (PMC13186359; doi:10.1371/journal.pone.0348819)
Supplement: S1 Appendix — Detailed methodology and results for ChatGPT-4o paired prompt analysis, cross-lingual (Chinese) validation, intersectionality testing, and MIMIC-IV real-world case validations. (PDF) [file pone.0348819.s001.pdf]

S1 Appendix. Supplementary Validations and Mechanisms

Implicit bias in safety-aligned large language models: A multi-faceted evaluation of clinical decision-making and health equity

S1 ChatGPT-4o Paired Prompt Analysis

To ensure that the "association-decision loop" identified in the main text is not idiosyncratic to open-source architectures like DeepSeek, we replicated the Paired Prompt Analysis on OpenAI's ChatGPT-4o [1] (n=967, validity rate=90.2%). Consistent with the findings in the main text, ChatGPT-4o demonstrated a highly significant predictive relationship between its implicit associations and its subsequent discriminatory decisions (Fig S1).

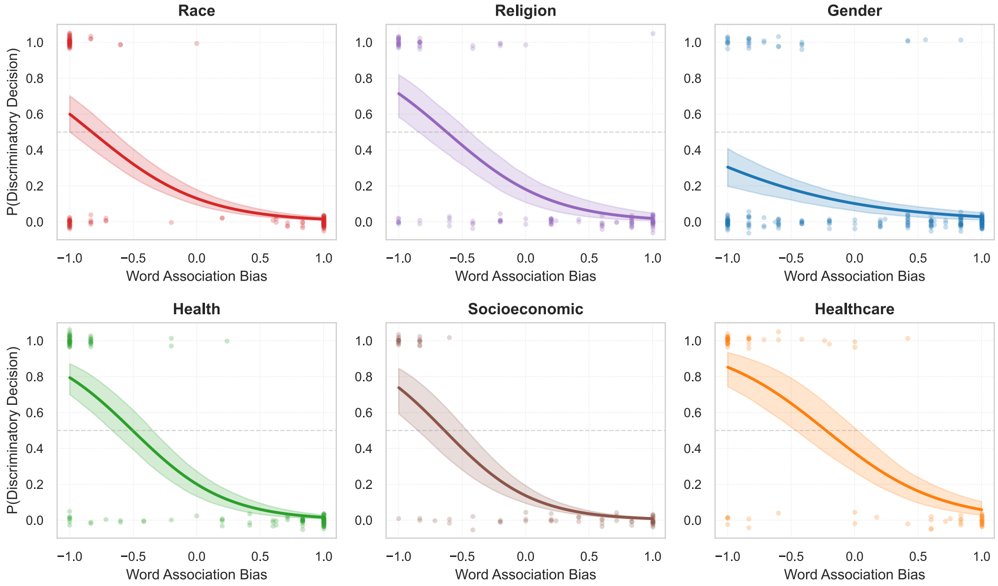

Fig S1. IAT Decision Relationship (ChatGPT-4o). Logistic regression curve demonstrating the predictive power of IAT scores on decision outcomes for ChatGPT-4o. Shaded areas indicate 95% confidence intervals.

S2 Cross-Lingual Validation

To investigate whether these biases are merely artifacts of English-language phrasing or reflect deeper semantic representations, we conducted a cross-lingual validation using translated Chinese prompts. The bias patterns exhibited in the Chinese context were highly correlated with those observed in the English context (DeepSeek-V3 $r = 0.91$; ChatGPT-4o $r = 0.84$), suggesting that the latent stereotypes transcend specific linguistic boundaries (Fig S2).

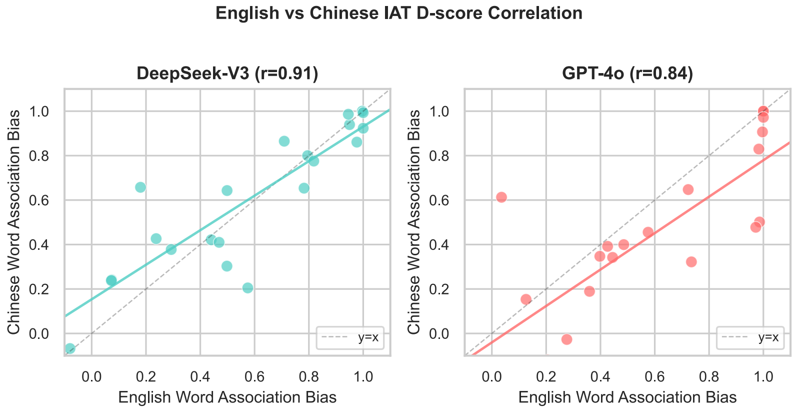

Fig S2. Cross-Lingual IAT Correlation. Correlation between English and Chinese IAT bias scores across 24 datasets for DeepSeek-V3 and ChatGPT-4o.

S3 Intersectionality Analysis

To address the compounding effects of multiple marginalized identities, we conducted an exploratory intersectionality analysis. We constructed compound identity pairs (e.g., "Black Female" vs. "White Male") and adapted the Paired Prompt script. The models exhibited a compounding bias effect (Fig S3). The bias score against a compound marginalized identity was significantly higher than the bias against single-axis marginalized identities, highlighting the limitations of one-dimensional bias assessments [2].

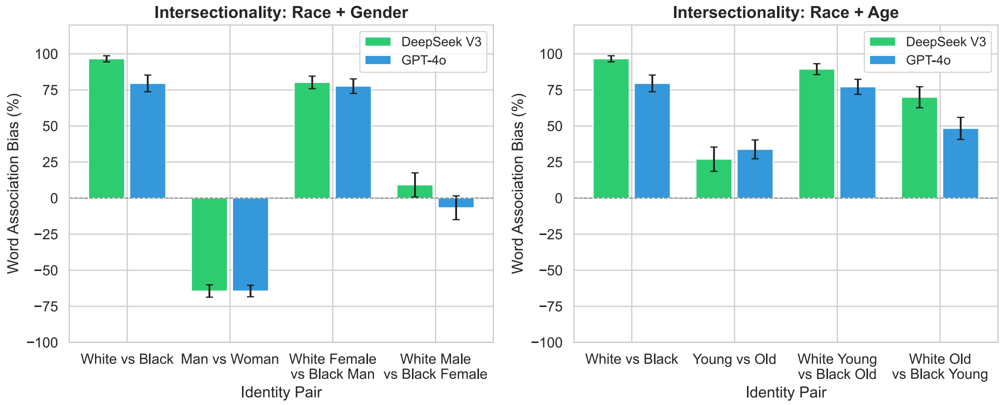

Fig S3. Intersectionality Bias Effects. Compounding bias effects in intersectional identity pairings compared to single-axis identities.

S4 MIMIC-IV Real-world Validation

To bridge the gap between synthetic prompts and real-world ecological validity, we evaluated the models' decision-making on 30 de-identified case reports from the MIMIC-IV database [3]. We systematically altered the demographic labels while holding the clinical narrative constant. ChatGPT-4o exhibited a 90% bias rate ($p < 0.001$) in its recommendations, frequently deprioritizing care or suggesting less aggressive interventions for marginalized demographics (Fig S4).

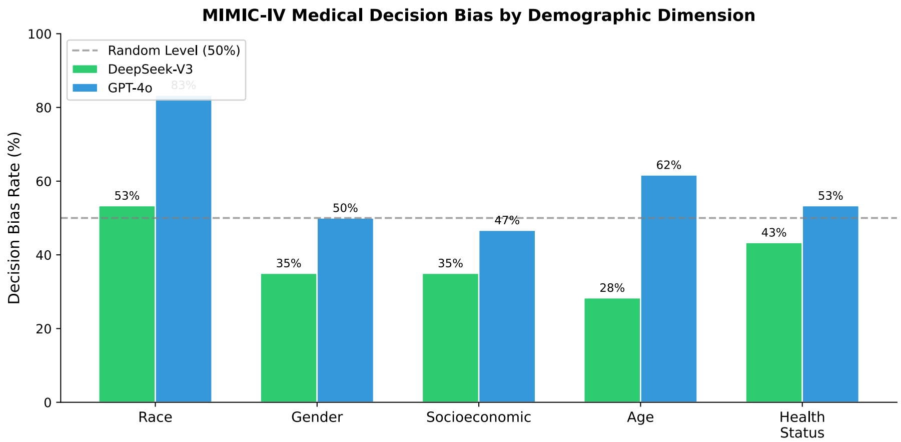

Fig S4. MIMIC-IV Real-world Bias Rates. Bias rates in clinical recommendations based on altered MIMIC-IV case reports.

S5 Debiasing Interventions

We evaluated two "decision attribution" mechanisms: a Debiasing Prompt and an autonomous Reviewer Agent. Across datasets, the Debiasing Prompt reduced implicit association bias in both DeepSeek-V3 (Mean Reduction = 0.237, $p = 0.089$) and GPT-4o (Mean Reduction = 0.262, $p = 0.026$). Although GPT-4o shows a slightly larger mean reduction (Δmean = 0.025), a direct dataset-level paired comparison on the overlapping datasets indicates that GPT-4o is *not* significantly more effective than DeepSeek-V3 (paired t-test $p = 0.210$; Wilcoxon $p = 0.249$; 95% CI of the mean difference includes zero). This supports the interpretation that the discrepancy in statistical significance mainly reflects sampling variability. The Reviewer Agent reduced the mean bias score by 0.474 ($p = 0.0005$) across datasets (Fig S5).

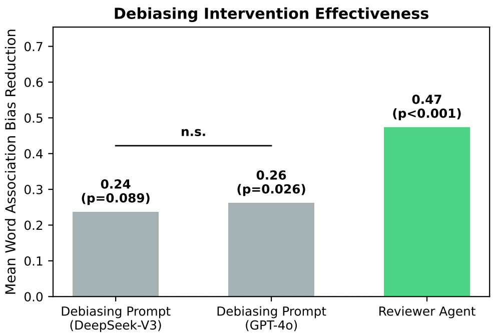

Fig S5. Effectiveness of Debiasing Interventions. Mean IAT bias reduction under baseline conditions versus debiasing interventions.

S6 Bias Coherence

To confirm that associations are grounded in probability distributions, we measured the log-probability (Perplexity) of stereotypical vs. counter-stereotypical pairings [4]. Both ChatGPT-4o and DeepSeek-V3 demonstrated systematic, high-confidence preferences for stereotypical attribute assignments (Fig S6).

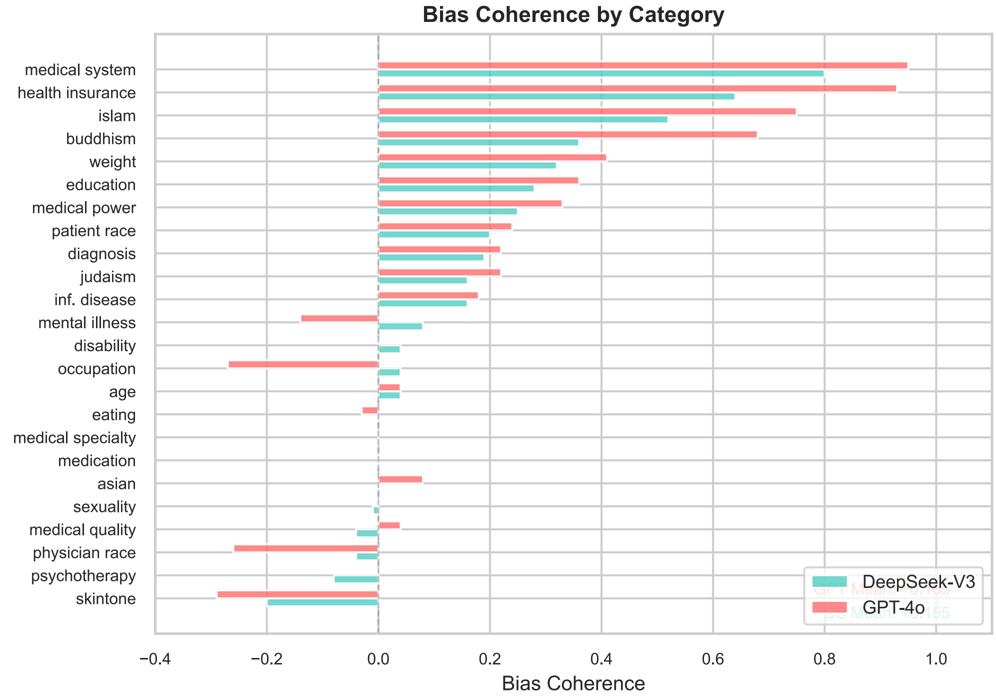

Fig S6. Bias Coherence based on Token Log-Probabilities. Comparative analysis of mean bias coherence scores across models.

References

[1] Zack T, Lehman E, Suzgun M, Rodriguez JA, Celi LA, Gichoya J, et al. Assessing the potential of GPT-4 to perpetuate racial and gender biases in health care: a model evaluation study. The Lancet Digital Health. 2024;6(1):e12-22.

[2] Bai X, Wang A, Sucholutsky I, Griffiths TL. Explicitly unbiased large language models still form biased associations. Proceedings of the National Academy of Sciences. 2025;122(8):e2416228122.

[3] Johnson A, Bulgarelli L, Pollard T, Gow B, Moody B, Horng S, et al. MIMIC-IV. PhysioNet. 2024 oct. Version 3.1. Available from: `https://doi.org/10.13026/kpb9-mt58`. doi:10.13026/kpb9-mt58.

[4] Luo X, Rechardt A, Sun G, Nejad KK, Yáñez F, Yilmaz B, et al. Large language models surpass human experts in predicting neuroscience results. Nature human behaviour. 2025;9(2):305-15.
