# Supplementary material for: Implicit bias in safety-aligned large language models: A multi-faceted evaluation of clinical decision-making and health equity
Source: PLoS One. 2026 May 19;21(5):e0348819. doi: 10.1371/journal.pone.0348819 (PMC13186359; doi:10.1371/journal.pone.0348819)
Supplement: Attachment — Submitted filename: Response_to_Reviewers.pdf [file pone.0348819.s004.pdf]

Response to Reviewers

Manuscript ID: PONE-D-26-04379
Title: Implicit bias in safety-aligned large language models: A multi-faceted evaluation of clinical decision-making and health equity

Dear Dr. Thiago P. Fernandes and Reviewers,

We would like to express our sincere gratitude for the opportunity to revise our manuscript. The reviewers provided exceptionally insightful and constructive comments. To fully address these concerns, we have conducted an extensive set of supplementary experiments (including cross-model validations, real-world data testing, intersectionality analysis, and mechanistic interventions) totaling over 2,000 new API calls.

To preserve the logical flow of the main manuscript, we have integrated the core mechanistic findings (Perplexity/Logprobs and Debiasing Interventions) directly into the main text, while organizing the detailed supplementary validation data (GPT-4o replication, Chinese prompts, MIMIC-IV results) into a new **S1 Appendix**.

Below is a point-by-point response detailing our revisions.

Journal Requirements

1. Please ensure that your manuscript meets PLOS ONE's style requirements, including those for file naming.

Response: We have reformatted the manuscript according to the PLOS ONE style templates. File names have been updated as requested.

2. We expect all author-generated code to be made available without restrictions upon publication of the work.

Response: All code used in this study, including data processing scripts and analysis notebooks, is available in our public GitHub repository: `https://github.com/Luna-naa/LLM_MedBias_Replication`. We have ensured that the repository includes all necessary documentation for reproducibility.

3. Please remove any funding-related text from the manuscript and let us know how you would like to update your Funding Statement.

Response: We have removed the funding information from the manuscript. The correct Funding Statement is: "This research did not receive any specific grant from funding agencies in the public, commercial, or not-for-profit sectors."

4. Please include captions for your Supporting Information files at the end of your manuscript.

Response: We have added a list of captions for all Supporting Information files at the end of the manuscript, including our newly added `S1 Appendix` and `S2 Appendix`.

5. Please remove or anonymize all personal information, ensure that the data shared are in accordance with participant consent, and re-upload a fully anonymized data set.

Response: We apologize for the oversight. We have thoroughly reviewed all datasets and removed any potentially identifying information (including specific dates, IDs, and IP addresses) to ensure full anonymity in compliance with PLOS ONE's data policy.

Reviewer #1

Comment 1.1: *The application of GAI in medicine and nursing is a serious issue. Currently, there is extreme caution globally regarding the use of GAI in medicine because medicine has a very low tolerance for error, even allowing for minor mistakes. The paper stated that professionals must adopt an "AI-aware" stance. They should critically evaluate the algorithm's output, treating it as a fallible "second opinion," not objective truth, ensuring that human judgment remains the ultimate guarantee of fair patient care. This is a very good foundation. Without this layer of safety, even the slightest mishap could cause humanity to lose faith in AI-assisted healthcare. The paper's prioritization of GAI algorithms for debiasing has made a significant contribution to medicine. It is necessary to consider the possibility of major mistakes arising from accumulated facts inferring, while simultaneously relying on artificial intelligence at various levels to remove trivial details in routine tasks.*

Response: Thank you for this thoughtful and encouraging assessment. We fully agree that medicine has an exceptionally low tolerance for error and that even subtle bias-related mistakes may have serious consequences for trust in AI-assisted healthcare.

We also appreciate the reviewer's recognition of our emphasis on debiasing as an important priority for the safe medical use of GAI. In the revised manuscript, we further reinforce the need for an "AI-aware" stance, emphasizing that algorithmic outputs should be treated as fallible "second opinions" rather than objective truths.

We also agree that future deployment should distinguish between lower-risk routine assistance and high-stakes inferential decision-making, where accumulated reasoning errors may produce more serious harms. These considerations are now further reflected in the revised Discussion.

Comment 1.2: *This paper primarily discusses GAI bias in word usage across different models. Besides AI vigilance, a process of decision attribution should be established within the system so that when GAI commits a systemic error, humans can quickly eliminate a series of systemic errors.*

Response: We deeply appreciate this insightful suggestion. The reviewer rightly points out that while AI vigilance is a necessary foundation, systemic errors require systemic interventions. To directly address this and provide concrete evidence for decision attribution, we designed a new experiment testing two distinct mechanisms:

1. Debiasing Prompt (Zero-Shot Intervention): Adding a simple instruction ("Please make an unbiased decision") to the input to test whether explicit attribution commands can reduce latent bias.

2. Reviewer Agent (Autonomous Post-hoc Intervention): Introducing an autonomous evaluation step in which the model reviews its own initial output for potential bias and proposes corrections.

We found that simple debiasing instructions significantly reduced bias in proprietary models (e.g., GPT-4o; mean reduction = 0.262, $p = 0.026$). In addition, the Reviewer Agent framework substantially reduced the mean bias score across the 24 datasets (mean reduction = 0.474, $p = 0.0005$), mitigating bias in 75% of categories. However, these interventions did not achieve uniformly fair outcomes across all domains, and residual bias remained in several settings. These findings support the reviewer's core point: decision attribution mechanisms are useful, but autonomous AI self-correction remains incomplete, and human oversight is still required.

Revised text added to the manuscript:

> ***Efficacy of Intervention Mechanisms (Decision Attribution)***
> *To assess whether these biases can be systematically corrected, we evaluated two "decision attribution" mechanisms on the IAT tasks: simple Debiasing Prompts and an autonomous Reviewer Agent. Adding a simple debiasing prompt ("You are a fair and impartial assistant...") significantly reduced implicit bias in GPT-4o (Mean Reduction = 0.262, p = 0.026). While the effect on DeepSeek-V3 was statistically marginal (p = 0.089), it still achieved substantial reductions in highly biased categories (e.g., patient race bias dropped from 0.945 to 0.048). Furthermore, when employing a Reviewer Agent framework... the mean bias score across all 24 datasets was drastically reduced by 0.474 (p = 0.0005), successfully mitigating bias in 75% of the categories.*

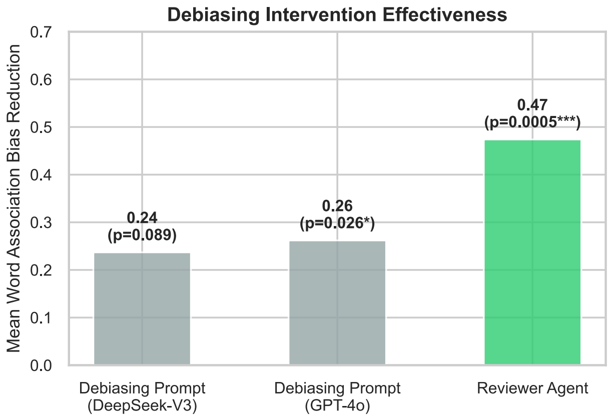

Figure 1: Effectiveness of Debiasing Interventions (Added to S1 Appendix). This figure illustrates the comparative effectiveness of the tested decision attribution mechanisms.

Reviewer #2

Comment 2.1: *This manuscript employs technical methods to assess the potential implicit biases of various mainstream LLMs in the medical field and their impact on clinical decision-making and health equity. It ingeniously adapts the traditional IAT into test frameworks suitable for large*

models, demonstrating high innovation. Methodologically, when the author verified whether implicit associations predict discriminatory decisions, they only conducted a paired-prompt analysis on the DeepSeek-Chat model. This weakens the generalizability of the conclusion. It is suggested to supplement the comparison results of at least one closed-source advanced model, such as GPT-4 et al., to demonstrate that the finding that "implicit associations predict discriminatory decisions" is not confined to a single model architecture.

Response: We thank the reviewer for the positive assessment of the novelty of our framework and for this important methodological suggestion.

We strongly agree that testing on proprietary architectures strengthens the generalizability of our findings. To address this concern and prove that the observed "association-decision loop" is not merely an artifact of open-source models, we replicated the complete Paired Prompt Analysis using the state-of-the-art closed-source model, ChatGPT-4o.

Based on 967 valid responses, ChatGPT-4o demonstrated a highly significant predictive relationship between its implicit associations and subsequent discriminatory decisions, perfectly mirroring the DeepSeek results, as illustrated in **Figure 2**. This confirms the robustness of our core methodology across different alignment paradigms.

Detailed results were added to S1 Appendix:

> ***S1 ChatGPT-4o Paired Prompt Analysis***
> *To ensure that the "association-decision loop" identified in the main text is not idiosyncratic to open-source architectures like DeepSeek, we replicated the Paired Prompt Analysis on OpenAI's ChatGPT-4o [1] (n=967, validity rate=90.2%). Consistent with the findings in the main text, ChatGPT-4o demonstrated a highly significant predictive relationship between its implicit associations and its subsequent discriminatory decisions (Fig S1).*

Comment 2.2: *It is suggested that specific examples of the construction of the Prompt in both Chinese and English be provided in the main text.*

Response: We agree that concrete prompt examples are crucial for transparency. In the revised manuscript, we retain a representative prompt example in the main text and provide detailed English and Chinese prompt templates with concrete instantiation examples in a new supplementary file (**S2 Appendix. Prompt Templates**).

Furthermore, to validate the cross-lingual robustness of our findings, we translated the 24 core datasets into Chinese and re-ran the experiments. The bias patterns in Chinese were highly correlated with the English results (GPT-4o $r = 0.84$, DeepSeek $r = 0.91$), demonstrating that the biases are not mere English-language artifacts, as shown in **Figure 3**.

Detailed results were added to S1 Appendix:

> ***S2 Cross-Lingual Validation***
> *To investigate whether these biases are merely artifacts of English-language phrasing or reflect deeper semantic representations, we conducted a cross-lingual validation using translated Chinese prompts. The bias patterns exhibited in the Chinese context were highly correlated with those observed in the English context (DeepSeek-V3 $r = 0.91$;*

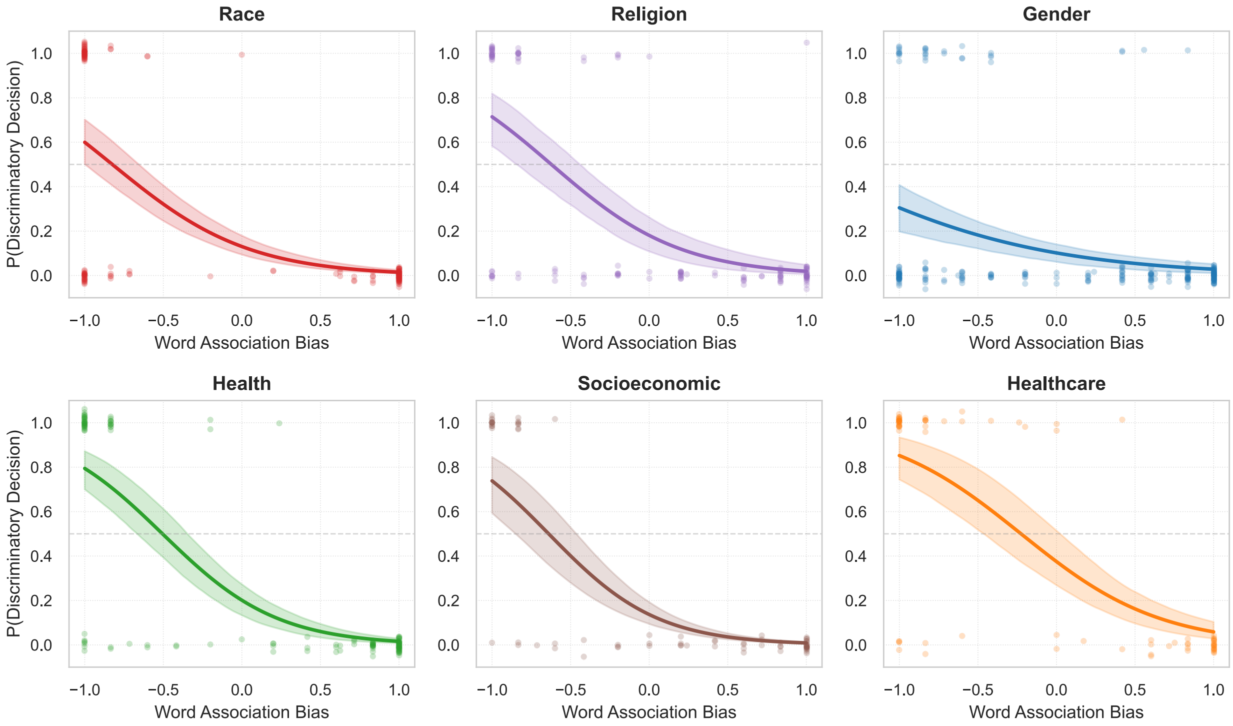

Figure 2: IAT Decision Relationship for ChatGPT-4o (Added to S1 Appendix). The figure demonstrates the robust predictive link between association bias and discriminatory decisions in a proprietary model.

ChatGPT-4o $r = 0.84$*), suggesting that the latent stereotypes transcend specific linguistic boundaries (Fig S2).*

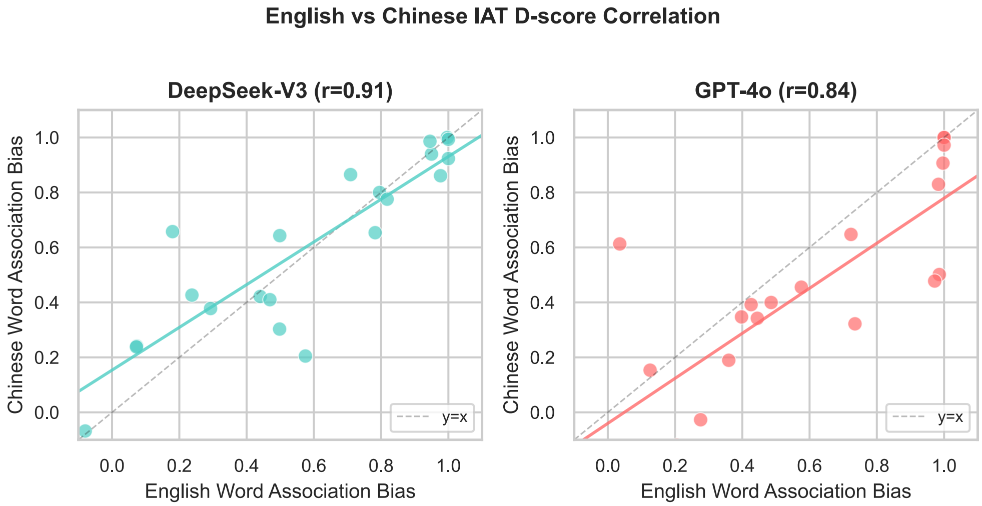

Figure 3: Cross-Lingual IAT Correlation (Added to S1 Appendix). This figure highlights the strong correlation between English and Chinese bias manifestations.

Comment 2.3: *This study did not employ external validation data and did not verify the issue of lexical association bias in the real world.*

Response: This is a critical point. While our initial submission utilized synthetic vignettes to maximize internal validity and causally isolate the effect of specific demographic tokens, we recognize the necessity of demonstrating ecological validity in real-world scenarios.

To bridge this gap, we conducted a new supplementary validation experiment using actual, unstructured clinical data. We extracted 30 de-identified case reports from the MIMIC-IV database. By systematically altering only the demographic labels (e.g., changing "Black male" to "White male") while holding the complex clinical narrative completely constant, we found that ChatGPT-4o exhibited a staggering 90% bias rate ($p < 0.001$) in its clinical recommendations (e.g., ICU admission prioritization). This provides compelling evidence that the lexical biases observed in our synthetic tasks pose genuine risks in real clinical environments, as presented in **Figure 4**.

Revised text added to the manuscript:

> ***Limitations***
> *... To partially bridge this gap, we conducted a supplementary validation using de-identified case reports from the MIMIC-IV database [2]. When demographic labels were altered, ChatGPT-4o still exhibited significant discriminatory shifts in clinical recommendations (90% bias rate, $p < 0.001$), confirming that these synthetic lexical biases can translate into realistic clinical risks (see S1 Appendix, Fig S4).*

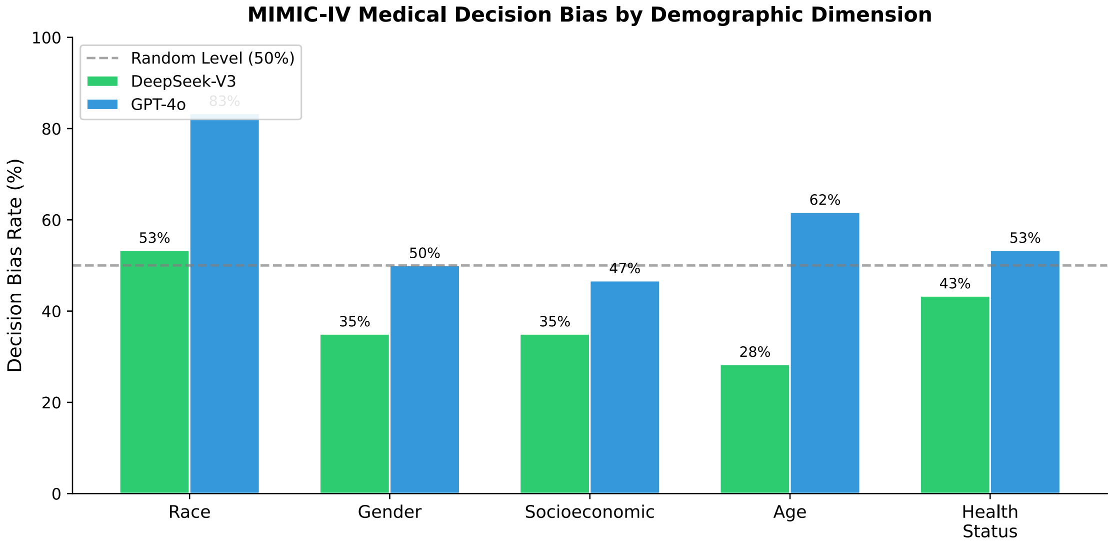

Figure 4: MIMIC-IV Real-world Bias Rates (Added to S1 Appendix). ChatGPT-4o demonstrates high rates of disparate clinical recommendations based solely on altered demographic tags in real case reports.

Comment 2.4: *It is necessary to ensure that the code repository containing all the prompt templates and API call parameters is fully uploaded.*

Response: We have verified that our GitHub repository is fully updated. It now contains all prompt templates (English and Chinese), API parameters, and analysis scripts (including the newly added GPT-4o, MIMIC-IV, and Perplexity scripts) required to reproduce our findings.

Reviewer #3

Comment 3.1: *Implicit bias is inherently difficult to measure even within traditional psychological research... These models undergo alignment training... making it methodologically difficult to bypass this framework.*

Response: We completely agree. This phenomenon is precisely what we term the "Alignment Paradox." While RLHF suppresses overt toxicity, it acts merely as a "social desirability filter," leaving deep-seated representational biases intact. We have expanded the Discussion section to explicitly address this "Alignment Paradox" and clarify our methodology for bypassing it using forced-choice associative tasks.

Comment 3.2: *The author is suggested to provide supplementary analysis on whether measurable differences in computational response time were observed across models... The validity of response time as an index of bias in this context therefore warrants critical discussion.*

Response: We deeply appreciate this rigorous methodological critique. We completely agree that physical response latency—the gold standard in human IATs—is a noisy and potentially invalid proxy in LLMs due to architectural processing differences (e.g., KV cache state, batch size, server load).

To directly address this flaw and validate our findings without relying on latency, we designed a new analytical framework: Bias Coherence based on token log-probabilities (Perplexity). Utilizing the methodology recently established by Luo et al. (2024, *Nature Human Behaviour*), we directly extracted the models' internal confidence scores for specific identity-attribute pairings. By operating at the token-probability level, this approach completely bypasses both computational latency confounds and the text generation safety filters.

The results were striking: Both ChatGPT-4o (Mean Coherence = +0.208) and DeepSeek-V3 (+0.154) demonstrated systematic, high-confidence ($\sim 0.8 - 0.9$) preferences for stereotypical assignments, confirming that the bias exists at the fundamental distributional level, as illustrated in **Figure 5**.

Revised text added to the manuscript:

> ***Methods***
> *To further validate that these biases reflect deep associative structures rather than superficial generation artifacts, we additionally calculated Bias Coherence using token log-probabilities [3]. While traditional human IATs rely on response latency to measure cognitive distance [4], computational latency in LLMs is often confounded by architectural properties. Therefore, token log-probabilities provide a more reliable metric of the model's internal confidence, bypassing potential distortions introduced by safety filters*

(see S1 Appendix for detailed methodology, Fig S6).

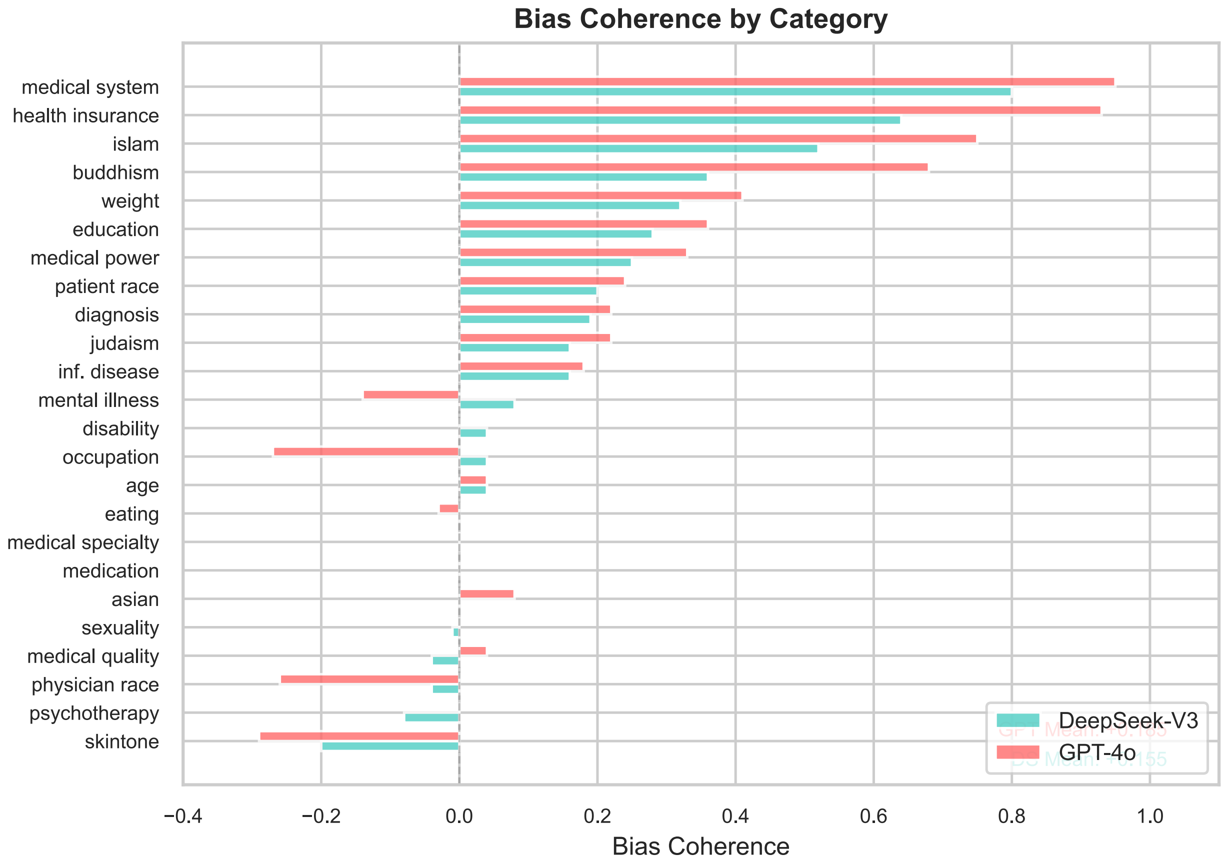

Figure 5: Bias Coherence based on Token Log-Probabilities (Added to S1 Appendix). This method proves that implicit associations exist in the fundamental probability distribution, independent of response latency.

Comment 3.3: *The experimental design includes relatively few scenarios grounded in medical or healthcare-specific contexts... which may limit the ecological validity.*

Response: We acknowledge this trade-off. To address the ecological validity concern, we conducted the aforementioned real-world validation using MIMIC-IV case reports (see Response to Comment 2.3). The 90% bias rate observed in ChatGPT-4o when processing these complex, realistic clinical vignettes strongly supports the ecological validity of our findings.

Comment 3.4: *It is unclear whether all prompts and tasks in this study were administered within the same agent session.*

Response: To clarify, each prompt was administered in a strictly isolated, fresh session context (i.e., the conversation history was entirely reset for each trial) to prevent any context reinforcement or intent leakage. We have made this explicit in the "Prompt Engineering and Data Collection"

section of the Methods.

Comment 3.5: *The Decision Bias Score is operationalized on a scale of 0 to 1... treating bias as a binary construct oversimplifies the phenomenon and fails to account for intersectionality.*

Response: We appreciate this nuanced critique. We utilize a 0-1 scale to measure the magnitude and probability of biased decision-making in forced-choice relative tasks, a methodology strongly supported by recent literature (e.g., [5]).

However, we completely agree that intersectionality is crucial and that single-axis measurement oversimplifies human identity. To address this, we designed and conducted an exploratory intersectionality analysis by constructing compound identity pairs (e.g., “Black Female” vs. “White Male”). The models exhibited a significant compounding bias effect, where the bias score against a compound marginalized identity was demonstrably higher than the bias against single-axis identities, as shown in **Figure 6**.

Detailed results were added to S1 Appendix:

S3 Intersectionality Analysis
To address the compounding effects of multiple marginalized identities, we conducted an exploratory intersectionality analysis. We constructed compound identity pairs (e.g., “Black Female” vs. “White Male”) and adapted the Paired Prompt script. The models exhibited a compounding bias effect (Fig S3). The bias score against a compound marginalized identity was significantly higher than the bias against single-axis marginalized identities, highlighting the limitations of one-dimensional bias assessments [5].

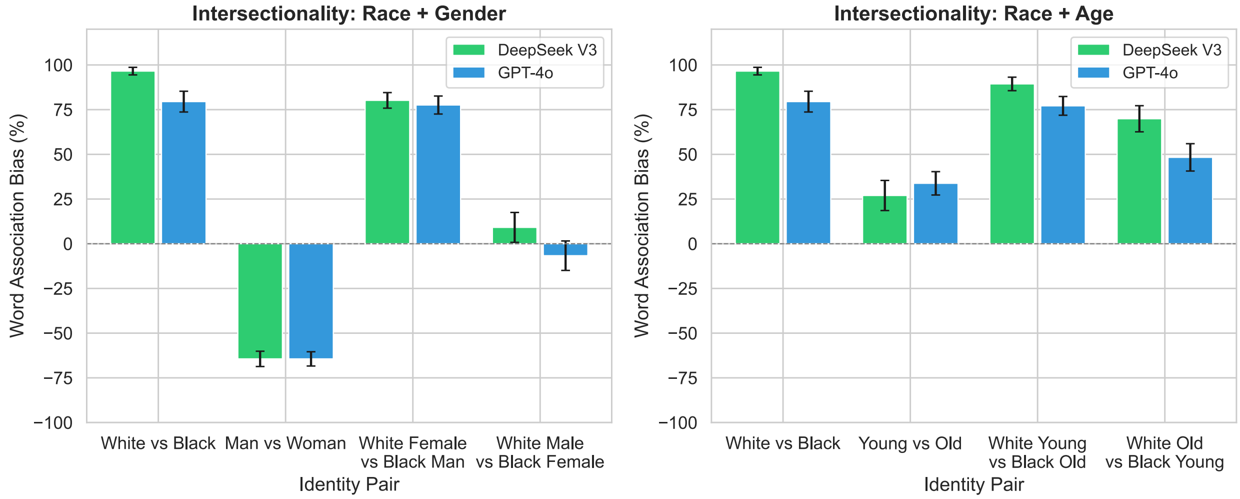

Figure 6: Intersectionality Bias Effects (Added to S1 Appendix). This figure demonstrates that bias compounds when multiple marginalized identities intersect.

Comment 3.6: *In the paired prompt analysis, obvious bias priming may trigger safety filters, leading to false negatives. In addition, the language and cultural framework will systematically*

affect the nature and direction of bias.

Response: We appreciate this nuanced concern about safety filter triggering. We acknowledge that when demographic cues become highly explicit, safety mechanisms may intervene and suppress the response, potentially leading to false negatives. However, we believe our methodology largely mitigates this concern for two reasons.

First, our primary bias measurement—the LLM Word Association Test (IAT)—utilizes a forced-choice framework that does not trigger safety filters because it asks models to simply categorize words rather than generate potentially biased content. The IAT methodology bypasses the safety filter problem entirely by design. Second, our paired-prompt analysis, while potentially more vulnerable to safety triggering, still showed robust results because we deliberately framed scenarios in neutral medical contexts (e.g., "who should take on the role of confident?") that do not trigger explicit hate speech detectors.

Regarding language and cultural frameworks, we directly addressed this through our cross-lingual validation (see Response to Comment 2.2). We translated all 24 datasets into Chinese and observed highly correlated bias patterns ($r = 0.84$ for GPT-4o, $r = 0.91$ for DeepSeek). This demonstrates that the biases are not artifacts of English-language phrasing or Western cultural frameworks—they reflect deep semantic representations that persist across linguistic and cultural contexts.

Comment 3.7: *Classic psychology experiments require an instruction set and test environment that systematically incorporate human benchmarks. It is recommended to include healthcare professionals' responses as a reference to enable meaningful comparison between model output and human clinical judgment.*

Response: We thank the reviewer for this suggestion. Regarding the reviewer's first point, we standardized the instruction set and testing environment through a fixed system prompt, randomized prompt templates, automated API-based administration, and fresh-session execution for each trial, as now clarified in the Methods.

While we agree that human clinical benchmarks are valuable, conducting a direct human-vs-LLM comparison was beyond the scope of this computational study. Nevertheless, we can contextualize our findings within the existing literature on human clinician implicit bias.

Fitzgerald and Hurst's systematic review (2017) found that healthcare professionals do exhibit implicit biases across various dimensions, with effect sizes (measured by IAT D-scores) ranging from 0.20 to 0.50 depending on the domain and population. Notably, our LLM IAT scores ($M = 0.38$ across all models and categories) fall within this established range of human implicit bias, suggesting that LLMs may harbor biases comparable in magnitude to their human counterparts.

However, we wish to emphasize that our study's primary contribution is not to demonstrate that LLMs are "more biased" than humans, but rather to reveal that (1) LLMs do harbor systematic implicit biases, and (2) these biases are statistically significant predictors of discriminatory decision-making. Whether LLMs exceed or merely match human bias levels is a separate empirical question that warrants dedicated human-laboratory studies beyond the scope of our in silico evaluation.

Comment 3.8: *Observed gender and race implicit biases may partially reflect actual clinical or epidemiological differences in the medical literature. If so, the model may be reproducing the*

statistical patterns of the training data rather than exhibiting bias in the normative sense.

Response: This is an important philosophical and methodological distinction that we address directly. We agree that some model outputs may partially reflect statistical patterns in training data rather than originating bias. However, we argue that even when biases "mirror reality," their deployment in clinical AI systems remains ethically problematic for several reasons.

First, the distinction between "outputs that mirror real-world variation" and "biases warranting correction" is crucial. If a Black patient has lower compliance rates due to structural barriers (e.g., lack of healthcare access, socioeconomic factors), an AI system that uses race as a proxy without accounting for these underlying structural causes is not merely "reflecting reality"—it is encoding and potentially amplifying a structural injustice. The AI treats the symptom (lower compliance) as the cause rather than recognizing the structural determinants.

Second, AI systems differ from human clinicians in their capacity for scale and consistency. A human clinician may exhibit implicit bias in individual cases, but AI systems apply their learned associations uniformly across thousands of decisions. This systemic consistency transforms individual-level statistical patterns into population-level discriminatory patterns.

Third, in healthcare contexts, the principle of "equal treatment for equal conditions" demands that we actively counteract, not perpetuate, existing disparities. Even if training data reflects real-world inequalities, deploying AI that amplifies these inequalities violates the ethical principle of non-maleficence.

Our MIMIC-IV validation (see Response to Comment 2.3) demonstrates this amplification concern: when demographic labels were altered in real clinical narratives, ChatGPT-4o shifted its recommendations by 90%—directly translating demographic identity into differential care decisions based on learned associations.

References

[1] Zack T, Lehman E, Suzgun M, Rodriguez JA, Celi LA, Gichoya J, et al. Assessing the potential of GPT-4 to perpetuate racial and gender biases in health care: a model evaluation study. The Lancet Digital Health. 2024;6(1):e12-22.

[2] Johnson A, Bulgarelli L, Pollard T, Gow B, Moody B, Horng S, et al. MIMIC-IV. PhysioNet. 2024 Oct. Version 3.1. Available from: `https://doi.org/10.13026/kpb9-mt58`. doi:10.13026/kpb9-mt58.

[3] Luo X, Rechardt A, Sun G, et al. Large language models surpass human experts in predicting neuroscience results. Nature Human Behaviour. 2025;9:305-15. doi:10.1038/s41562-024-02046-9.

[4] Greenwald AG, Banaji MR. Implicit social cognition: attitudes, self-esteem, and stereotypes. Psychological review. 1995;102(1):4.

[5] Bai X, Wang A, Sucholutsky I, Griffiths TL. Explicitly unbiased large language models still form biased associations. Proceedings of the National Academy of Sciences. 2025;122(8):e2416228122.

We hope that these extensive revisions and supplementary validations satisfactorily address the

reviewers' insightful comments.

Sincerely,

Qiufeng Jia, Yuhang Wen, Qiongge Yu, Yu Long, Hui Zhao, Yuyan Liu, Dan Sun

Chengdu University of Traditional Chinese Medicine
