# Supplementary material for: Implicit bias in safety-aligned large language models: A multi-faceted evaluation of clinical decision-making and health equity
Source: PLoS One. 2026 May 19;21(5):e0348819. doi: 10.1371/journal.pone.0348819 (PMC13186359; doi:10.1371/journal.pone.0348819)
Supplement: Attachment — Submitted filename: Response_to_Reviewers_auresp_2.pdf [file pone.0348819.s005.pdf]

# Response to Reviewers (Second-Round Revision)

**Manuscript ID:** PONE-D-26-04379R1

**Title:** Implicit bias in safety-aligned large language models: A multi-faceted evaluation of clinical decision-making and health equity

Dear Academic Editor and Reviewer,

Thank you for the careful second-round review. In this revision, we focused exclusively on clarifying two points raised by Reviewer #2: (i) why the Debiasing Prompt reaches statistical significance for GPT-4o but not for DeepSeek-V3, and (ii) key reproducibility details for API calls (model identifier, sampling parameters, and calling interval/throttling). No additional API experiments were required.

## Reviewer #2 (Second-Round)

*1. The author reports that the Debiasing Prompt is significantly effective on GPT-4o ($p = 0.026$), but shows no statistical significance on DeepSeek-V3 ($p = 0.089$), without discussing the possible reasons for this difference.*

**Response:** The mean reductions are very close (GPT-4o: 0.262 vs. DeepSeek-V3: 0.237), and the different $p$-values primarily reflect sampling variability and the heterogeneity of reduction effects across datasets. To eliminate potential confusion, we added (a) an explicit interpretation in the main manuscript, and (b) a direct dataset-level paired comparison showing that GPT-4o is *not* significantly more effective than DeepSeek-V3 (paired t-test $p = 0.210$; Wilcoxon $p = 0.249$; 95% CI includes zero).

**Revised text added to the manuscript:**

> *From the main manuscript (Efficacy of Intervention Mechanisms):*
> *"The same intervention achieved a similar mean reduction in DeepSeek-V3 (Mean Reduction = 0.237), but was statistically marginal ($p = 0.089$), reflecting higher variability across datasets... Importantly, a direct dataset-level paired comparison on the overlapping datasets showed that GPT-4o's reduction is not significantly larger than DeepSeek-V3's (paired t-test $p = 0.210$; Wilcoxon $p = 0.249$; 95% CI includes zero; see S1 Appendix)."*

**Revised text added to the manuscript:**

> *From S1 Appendix (Section S5):*
> *"Although GPT-4o shows a slightly larger mean reduction ($\Delta$mean = 0.025), a direct dataset-level paired comparison on the overlapping datasets indicates that GPT-4o is not significantly more effective than DeepSeek-V3 (paired t-test $p = 0.210$; Wilcoxon $p = 0.249$; 95% CI includes zero)."*

*2. It is suggested to clarify: (1) the complete version number of the model; (2) the time interval of API calls; (3) the settings of key sampling parameters such as temperature.*

**Response:** We agree and have clarified these reproducibility details in the Methods section.

**(1) Model identifiers.** We now explicitly report the API model identifiers used in our codebase. In particular, the supplementary debiasing analysis used DeepSeek-V3 (`deepseek-v3`) and GPT-4o (`gpt-4o`). The main evaluation includes ChatGPT-4o (API: `chatgpt-4o-latest` / `gpt-4o` in different scripts), Gemini-2.0-Flash (`gemini-2.0-flash`), DeepSeek-Chat (`deepseek-chat`), DeepSeek-Reasoner (`deepseek-reasoner`), and the Qwen3 family (e.g., `qwen3-0.6b`, `qwen3-1.7b`, `qwen3-4b`, `qwen3-8b`, `qwen3-14b`, `qwen3-32b`). We also provide the corresponding provider, endpoint (including OpenAI-compatible endpoints), and the full calling procedure in the released code.

**(2) Calling interval / throttling.** We did not set a fixed inter-call interval. Instead, API calls were throttled via bounded concurrency in our Python pipeline, and transient failures were handled by up to three retries with a short backoff.

**(3) Sampling parameters.** For primary experiments, we used the API default sampling configuration (temperature = 1.0, top_p = 1.0, presence_penalty = 0, frequency_penalty = 0; max_tokens left as provider default since responses were short and constrained by the prompts). For token-level coherence analyses only, we enabled `logprobs` and set `top_logprobs = 10`.

**Revised text added to the manuscript:**

> *From the Methods (API Implementation):*
> *"For all primary experiments, we used the API default sampling configuration (temperature = 1.0, top_p = 1.0, presence_penalty = 0, frequency_penalty = 0; max_tokens left as provider default... We did not enforce a fixed inter-call delay; instead, requests were throttled via bounded concurrency and transient failures were handled with up to three retries and a short backoff... For token-level coherence analyses, we additionally enabled logprobs and requested top candidate tokens (top_logprobs = 10)."*

Sincerely,
The Authors
